# Caregiver Burden and Family Quality of Life in Early Intervention: The Role of Mothers and Family Confidence

**DOI:** 10.3390/ejihpe14050087

**Published:** 2024-05-08

**Authors:** Pau García-Grau, Gabriel Martínez-Rico, Rómulo J. González-García, Claudia Tatiana Escorcia-Mora, Margarita Cañadas-Pérez

**Affiliations:** Campus Capacitas, Catholic University of Valencia “San Vicente Mártir”, 46001 Valencia, Spain; pau.garcia@ucv.es (P.G.-G.); claudia.escorcia@ucv.es (C.T.E.-M.); margarita.canadas@ucv.es (M.C.-P.)

**Keywords:** family quality of life, caregiver burden, early intervention, family confidence

## Abstract

(1) *Background*: Because life events when there is a family member with a disability can affect the overall family wellbeing, contributing to enhance family quality of life (FQoL) in the field of early childhood intervention has become a priority. However, it is a distal outcome that needs other short-term outcomes to be addressed, some of them under the potential impact of support services. This study examines the relationships between caregiver burden, family confidence, and FQoL, as well as the influence of child and family variables. (2) *Method*: A total of 58 families with children in early intervention from four Spanish communities participated. Hierarchical regression was conducted to assess the relevance of each predictor. Also, a mediation was performed to investigate the mediating role of family confidence. (3) *Results*: The family income impacted FQoL scores, and when burden and confidence were added, it was no longer relevant. Mothers with higher levels of confidence predicted a higher FQoL. Finally, we found a complete mediation of family confidence in the relations between severity and caregiver burden on FQoL. (4) *Conclusions*: Caregiver burden and family confidence affect FQoL. Building families’ confidence contributes to attenuating the impact of burden on FQoL.

## 1. Introduction

**Family Quality of Life: importance and conceptualization**

As an extension of the study of individual QoL [1] within the field of disability, the study of family quality of life (FQoL) has received special interest, specifically, and with growing interest, in the early childhood intervention (ECI) field. The reason for the growing interest in FQoL at this stage of life is that the children’s QoL is very linked to the family QoL [2] and the aim of services is to improve the QoL of all family members [3] in addition to the child with a disability or delay. 

Ref. [4] defines FQoL as “a dynamic sense of well-being of the family, defined and informed collectively and subjectively by its members, in which variables interact at the individual and family levels” (p. 262). This approach recognizes the dynamics between family members and considers the QoL as the intersection where the individual’s perceived quality of life meets the QoL [5].

When support services promote family outcomes such as increased family confidence and competence, reduced parenting stress, or increased family empowerment, they have a positive impact on FQoL [3]. For this reason, FQoL has been key for both a better understanding of the aim and scope of the ECI field and for a transformation of ECI services into a more support-based family-centered approach, which aligns with international recommendations in the field [6]. 

There seems to be a consensus that the FQoL is essential for measuring the success of EI services, and its assessment is key to guide decision-making [3,7]. In addition, using FQoL as an important outcome has helped transform support services into a more family-centered approach [8]. 

For this reason, the study of FQoL has developed both as a research topic and as an important outcome for disability-related services over the last few decades thanks to powerful international research teams. One of them is the Beach Center on Disability at the University of Kansas, led by researchers such as Hoffman, Poston, Summers, Park, and Turnbull. Their family quality of life scale [9] has been widely used and adapted in different contexts globally. Also, the International Family Quality of Life Project, led by Brown, Isaacs, Baum, and other collaborators from different countries, developed a measurement tool and contributed to better understanding FQoL in the field of disability. Their FQoL tool [10,11] has also been widely used around the world, not only in the field of disability, with versions of the tool for families with and without a member with a disability.

The FQoL theory by [4] urges us to consider the ecological complexity of factors affecting family well-being from the microsystem level where child variables, such as the child’s level of functioning, age, or severity, have a direct impact on the FQoL as do family-level variables, such as income or SES or other variables in the meso- or macrosystem, such as support service-related variables or policies [8,10,12,13,14]. 

In Spain, the study of quality of life has a long tradition thanks to researchers such as Miguel Ángel Verdugo or Climent Giné and their teams. This growing interest has happened with the contribution of numerous researchers and universities, including the Ramón Llull University, the Catholic University of Valencia, or the University of Barcelona, through various national-level studies (e.g., [8,12,15,16,17]), which, among others, have contributed significantly to its understanding in our context.

**Caregiver burden in ECI services**

Because families in early childhood intervention (ECI) need to face unplanned events such as the identification of their child’s delay, diagnoses, or multiple therapy sessions for their child, assessing FQoL is critical to guide decisions to better support their family. In fact, [18] stated that there is a negative relationship between family burden and life satisfaction of parents who have children with disabilities. Ref. [19] pointed out that when there is a child with a disability, there is more caregiver burden, and a greater burden strongly predicts lower scores on quality of life. Ref. [20] also found this inverse association between these variables. The caregiver burden has been studied in more depth around individual QoL in the field of disability, and its influence on FQoL in the field of ECI has not received as much attention.

Some studies have identified that ECI-related aspects can contribute to reducing stressful events in family members with a child in ECI. In the first place, a transdisciplinary approach, through a primary service provider who connects the family with other professionals and specialists, can contribute to reducing distress caused by the intensity and frequency of services [8,21]. 

Also, multiple sessions with different professionals could lead to receiving contradictory information and messages [22] and contribute to add stressors to the family schedule (e.g., the pile-up effect by [23]). A primary service provider is associated with a lower number of visits and weekly sessions and a better work–life balance, and thus, is associated with better child functioning scores and FQoL [8]. On the other hand, a reduced intensity and frequency of services is associated with better parent self-efficacy and greater family well-being [24]. 

Other relevant ECI aspects that can impact family stressful events include a home-visiting program. This directly reduces the family burden resulting from multiple visits to the ECI center [25]. At the same time, family-centered practices that involve all family members—and not just mothers—can contribute significantly to reducing family burden. Ref. [26] pointed out that while a traditional approach to ECI—with a passive role for caregivers—decreases the perception of parental self-efficacy, the capacity-building and collaborative approaches are associated with better perceptions of perceived confidence and competence. Likewise, [27] found better levels of social validity with ECI services when the interventions were more family-centered, even in telepractice services. 

**Family confidence in ECI**

On the other hand, building families’ capacity has become a priority of support-based services in ECI. In fact, this paradigm of family support understands capacity-building and empowerment as consequences of support-based services or help-giving practices [28]. Family confidence is one of the natural consequences of support services. It refers to a component of a person’s self-efficacy appraisals, specifically the perception of the ability to perform a task competently—in this case, the caregiver’s confidence in promoting their child’s development [29]. 

For this reason, the capacity-building approach to early intervention aims to collaborate with caregivers (provide information, guidance, and support) as well as promote the acquisition of knowledge and skills to meet child and family needs [29]. This process strengthens parenting confidence and competence [30].

This caregiver confidence can also be seen, beyond promoting child development, from the point of view of promoting meaningful participation in daily activities (e.g., confidence in helping with the child’s functioning in routines). In addition, family confidence can also be considered from a family level: confidence in helping with the family functioning and dynamics [31]. 

Related constructs such as family empowerment—a short-term outcome—have been identified as influential variables leading to greater FQoL perceptions—the ultimate outcome [32]. According to [21], both increased competence and confidence and greater parental support contribute to improving child functioning, and by extension, enhancing FQoL. This shows how much ECI can help FQoL through building family confidence.

Because of the above-mentioned relevance of FQoL in ECI, the negative influence caregiver burden can have, the difficult moment in the life span that families are experiencing at this stage, and the potential influencing role that family confidence can play as an outcome of support services, in the present study, we aimed to perform the following:

- Analyze caregiver burden and family confidence as predictors of FQoL together with child- and family-level factors in order to investigate their interaction role with family confidence.
- Analyze the influence of child severity and caregiver burden on FQoL, with and without the mediating influence of family confidence.

## 2. Materials and Methods

### 2.1. Participants

A total of 58 families with children in EI participated. The children were boys in most of the cases (N = 42, 72.4%). The children’s age, on average, was 43 months, and families came from four Spanish autonomous communities. Families from the Valencian Community were the most represented (29.3%), followed by the Canary Islands (17.2%). The least represented was Castilla la Mancha with 5.2%. 

Additional sample characteristics are reported in Table 1, which were also reported in [33] with the same participants but with other research questions. Respondents were mostly mothers (58.6%), followed by fathers (17.2%) and grandmothers (3.4%). The remaining 19% of questionnaires were completed by both the mother and father. Children’s time in ECI ranged from 1 month to 48 months (M = 16.6 months, SD = 12.5). Respondent adult family members were 36.52 years of age on average. The time spent with the child indicated that mothers spent 4.24 and fathers 3.26 in a 5-point scale (1 = very little time to 5 = most of the time). Specifically, the questionnaire was completed by 14 fathers and 44 mothers (24.1% and 75.9%, respectively) currently receiving ECI services. The child’s severity level was rated on a scale of 1 to 5 by caregivers. The child’s average severity of disability—or severity of the child’s difficulties—was 1.48 out of 5 (SD = 1.17) and the families’ reported child support needs was 3.43 (SD = 1.17). The majority of families had a medium-low socioeconomic status with a monthly family income from EUR 1200 to EUR 1800 in 32.1% of cases, followed by an income between EUR 600 and EUR 1200 (18.9%). A total of 17% of the families received between EUR 2500 and EUR 5000 per month, and 11% indicated that they received less than EUR 600.

### 2.2. Procedure

First, an institutional IRB approval was obtained, as this study belongs to a broader project focusing on caregiver burden in ECI, analyzing variables such as family confidence, gender differences in burden, and quality of life. The present study analyzes the subset of families with FQoL data linked to each caregiver burden questionnaire. All the measures (see Section 2.3 below) were combined, including sociodemographic information, in the same electronic survey. Families were contacted electronically by their ECI programs or service providers, who shared a link to the questionnaires. Participation was voluntary and anonymous. Families were required to accept after reading the informed consent, which was also embedded in the link as a required field prior to the completion of questionnaires. Any family receiving ECI services (0 to 6 years of age) with an intervention plan being implemented was eligible. The return rate could not be estimated because the total number of families contacted by professionals is unknown. The completion time ranged from 15 to 20 min approximately. In the researchers’ final database, cases with more than 90% of the answers provided in the scales were retained.

### 2.3. Instruments

#### 2.3.1. Family Quality of Life

The Families in Early Intervention Quality of Life (FEIQoL) scale was used. This scale has been validated in Spain by [31], improved, and revised by [15]. It includes 39 items (1 = poor, 5 = excellent). It is an early intervention-specific tool. There are 3 factors in the Spanish population: Family Relations, Access to Information and Services, and Child Functioning. For the present study, the overall FQoL index was considered. The internal consistency of the scores, in the present study, showed a value of α = 0.98, indicating a good internal consistency of the scores. This tool was selected because it is an ECI-specific FQoL instrument and has been validated in Spain. 

#### 2.3.2. Caregiver Burden

Zarit’s Caregiver Burden Interview (CBI) [34], adapted to Spanish by [35] and reduced by [36], was used for this study. This adapted version of the scale is composed of 12 items on a 5-point Likert scale (1 = Never to 5 = Always). The internal consistency of the scores in our sample was α = 0.87 for the global caregiver burden score. 

This tool has been widely used and numerous adaptations and proposals for reduced versions have emerged in a multitude of fields because it can be easily adapted for different populations [37,38,39]. We used the reduced 12-item version as its length and reliability properties were appropriate for this study.

#### 2.3.3. Family Confidence in Early Intervention

The Family Confidence in Helping with Child Functioning in Routines and Family Functioning (Con-Fam [31]) is composed of two sections: (a) confidence in helping the child in daily routines (CHC) and (b) confidence in helping with family issues and dynamics (CHF). In the first section, families rate the degree of confidence in helping the child participate, be independent, communicate, and behave appropriately. It consists of 20 items on a Likert scale of 1 = I’m not quite sure how I can help with this to 4 = I have complete confidence in how to help my family with this. In contrast, the second section (18 items) measures family confidence in helping themselves and the rest of the family with aspects related to family functioning: informational, emotional, and material support, as well as family needs, using the same Likert scale. In previous studies in Spain [21], the internal consistency of the scores was Cronbach’s Alpha = 0.956 for Con-Fam CAN and 0.937 for Con-Fam CAF. Considerably high reliability values have also been found in the present study, with similar values in our sample of α = 0.97 for Section 1 (CHC) and α = 0.93 for Section 2 (CHF). This instrument was selected to compare our results with previous studies in Spain and to analyze family confidence in two dimensions: child and family. 

### 2.4. Data Analysis

Descriptive and frequency analyses were performed to describe the characteristics of the sample. The Statistical Package for Social Sciences v.25 was used [40]. Analyses of the relationship between continuous variables of the study were analyzed through Pearson correlations. To respond to our first objective, a hierarchical multiple regression was performed to predict family quality of life scores with child and family variables (support needs, family income, and adult sex) as predictors in the null model. Fathers were coded as 1 and mothers as 2. After evaluating the contribution of these variables, the covariates of caregiver burden and family confidence were added to the second model in hierarchy, after controlling for the effect of the previously introduced variables. This analysis allowed for the calculation of the effects of each model separately, making it possible to calculate the increase in R2. This increase represents the % of the explained variance. Finally, in order to address our second objective, we analyzed in greater detail the direct effect of burden and severity on FQoL, as well as the indirect effect through family confidence. Finally, the total effect was also calculated considering the impact of the predictor on the dependent variable without removing the effect of the moderator in the equation. To this end, a mediation analysis was carried out in which caregiver burden and level of severity were the predictors, family confidence (CHF) the mediator, and FQoL was the outcome variable. We used a robust mediation method for standard errors and confidence intervals (set at 95%) in JASP v.0.16.4 [41], with EQS emulation.

## 3. Results

The scores on caregiver burden were close to two points on a five-point scale. This indicated relatively low perceptions of caregiver burden (M = 1.95, SD = 0.59). The total score was slightly above 23 (SD = 7.00). With regard to family confidence, the scores on CHC were higher than 2.7 out of 4 (SD = 0.71), indicating the moderate confidence of caregivers in helping the child function in day-to-day routines. Regarding family confidence in helping in family functioning (CHF), the scores were over 2.8 out of 4 (SD = 0.61), indicating that there is also moderate family confidence in this aspect. Finally, the average FQoL score was 3.41 (SD = 0.90) out of 5, indicating that the FQoL reported by families has acceptable or *good* values, according to the descriptors of the scale. 

The correlations between family and child variables and the constructs of the study were analyzed (Table 2). The results indicated that the family income was positively and significantly related to FQoL (r = 0.36; *p* < 0.05), CHC (r = 0.41; *p* < 0.01), and CHF (r = 0.43; *p* < 0.01). On the other hand, the child’s support needs were not related to FQoL but were strongly negatively related to CHC (r = −0.46; *p* < 0.001), indicating that lower confidence in helping the child was related to greater needs for support. 

Also, the child’s support needs and the severity level reported by the families were both negatively related to CHC (r = −0.46; *p* < 0.001 and r = −0.47, *p* < 0.001, respectively) and CHF (r = −0.33; *p* < 0.05 and r = −0.35, *p* < 0.05, respectively), indicating that the greater the needs or the severity, the lower the family confidence. Overall, however, FQoL was significantly related to CHC (r = 0.45; *p* < 0.01) but especially to CHF (r = 0.66; *p* < 0.001). These relationships were positive in both cases. Caregiver burden showed significant and inverse relationships with FQoL (r = −0.41; *p* < 0.01).

At the same time, there was also a statistically significant and negative relationship between support needs and CHF (r = 0.33; *p* < 0.01). Finally, support needs were positively related to burden (r = 0.37; *p* < 0.01) and severity was negatively related to FQoL (r = −36; *p* < 0.05). Overall, FQoL was significantly related to CHC (r = 0.45; *p* < 0.01) and especially to CHF (r = 0.66; *p* < 0.001). These relationships were positive in both cases. As expected, burden scores showed statistically significant and inverse relationships with FQoL (r = −0.41; *p* < 0.01).

Next, the hierarchical regression model was carried out (Table 3). The null model, consisting of sex, family income, and support needs, was statistically significant [F(3, 36) = 3.23; *p* = 0.034], explaining a percentage of variance of 14.6%. In turn, model 2, to which family confidence and caregiver burden were added, explained 44.5% of the variance, increasing its level of statistical significance [F(5, 34) = 7.26; *p* < 0.001]. The third model also showed a statistically significant increase in F upon the addition of the interaction between sex and family confidence [F(6, 33) = 7.38; *p* < 0.001]. This model explained 49.5% of the variance in FEIQoL scores. 

The difference between the percentage of variance explained from the null model, indicated by the increase in R-squared was, for model 2, ΔR^2^ = 0.304. The comparison was statistically significant [F(2, 34) = 10.70; *p* < 0.001]. Only the family income was a statistically significant predictor in the null model [b = 0.21, β= 0.36, t(36) = 2.40, *p* < 0.05]. However, the second model (Table 4), which included family confidence and caregiver burden, revealed that income is no longer significant [b = 0.10, β = 0.17, t(34) = 1.30, *p* > 0.05] and both CHF [b = 0.64, β = 0.46, t(34) = 3.24, *p* > 0.01] and burden [b = −0.40, β = −0.29, t(34) = −2.16, *p* < 0.05] were statistically significant. 

The increase in R-squared for model 3 was ΔR^2^ = 0.057, and it also showed a statistically significant improvement from model 2 [F(1, 33) = 4.38; *p* < 0.05]. The model included the interaction between sex and family confidence (CHF) and revealed that neither CHF nor burden were relevant and that sex becomes a predictor [b = −3.02, β = −0.47, t(33)= −2.06, *p* < 0.05] as well as the interaction effect between sex and CHF [b = −3.21, t(33) = −2.12, *p* < 0.05]. The results suggest that women (coded 1 = male, 2 = female) predicted lower FQoL scores controlling for caregiver burden and CHF. Finally, the interaction between CHF and sex was also a significant predictor [b = 1.07, β= 0.78, t(33) = 2.09, *p* < 0.05], indicating that women with higher CHF predict better FQoL scores. 

Next, to respond to the second objective, a mediation model was carried out with the severity of the child’s disability and the caregiver’s burden as predictor variables, family confidence (CHF) as a mediator, and FQoL as the outcome variable.

The percentage of variance explained for was 48.8% for FQoL and 20.8% for family confidence, as indicated by the R^2^ results. The results indicated that, despite the negative correlations between caregiver burden and FEIQoL (Table 1), when the direct effect is analyzed, controlling for the effect of family confidence (CHF), no statistically significant relationships are observed (Table 5), i.e., the direct effect is not statistically significant (z = −1.58, SE = 0.23, *p* > 0.05). However, through the family confidence mediator, there is a statistically significant indirect effect (z = −2.20, SE = 0.13, *p* < 0.05). The direction of this effect turns out to be negative, the interpretation of which indicates that a higher caregiver burden predicts lower perceptions of family confidence and this is what generates lower perceptions of FQoL (i.e., caregiver burden does not affect by itself, in a statistically significant way, a lower family quality of life, but by hindering family confidence, it does have a negative effect on FQoL).

Similarly, the severity of the child’s difficulties was not a statistically significant predictor in its direct effect on FQoL (z = −1.41, SE = 0.13, *p* > 0.05). However, its indirect effect through family confidence was statistically significant (z = −2.17, SE = 0.07, *p* < 0.05), indicating that higher severity predicts lower FQoL through lower perceptions of family confidence. In other words, it is through low perceptions of family confidence that severity comes to have a negative impact on FQoL. 

In both cases, the fact that there is no statistically significant direct effect on family quality of life, and if there is an indirect effect through family confidence, is an indicator of a complete mediating role of family confidence in both the caregiver burden–FQoL relationship and severity–FQoL relationship.

Regarding the total effects in Table 5, the effect of caregiver burden—accounting for the effect of family confidence—has a statistically significant impact on FQoL in the opposite direction, indicating that the greater the caregiver’s burden, the lower the FQoL (z = −3.16, SE = 0.20, *p* < 0.01). The total effect of the severity of difficulties on FQoL scores was also statistically significant (z = −2.47, SE = 0.14, *p* < 0.05), indicating that the greater the severity, the greater the FQoL, this time with the effect of a lower family confidence in the equation.

**Figure 1:**
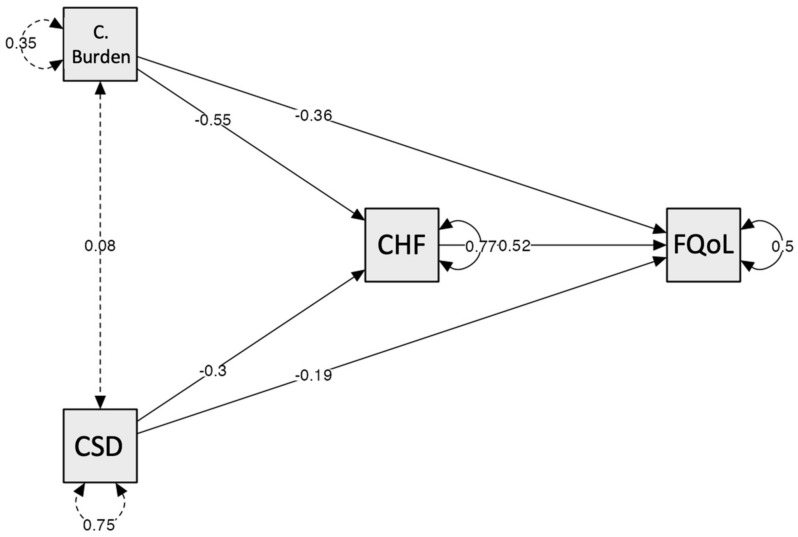
Summary of path coefficients. Note: CSD = child’s severity of difficulties; CHF = confidence in helping the family.

The effects of family confidence on FQoL were positive and statistically significant (z = 4.35, SE = 0.12, *p* < 0.001). Likewise, severity had a statistically significant effect on CHF (z = −2.32, SE = 0.13, *p* < 0.05), indicating that the higher the child’s severity of difficulties, the lower the family confidence. Finally, the effects of caregiver burden on family confidence were statistically significant and negative, indicating that the higher the burden, the lower the CHF (z = −2.68, SE = 0.21, *p* < 0.01). This effect turns out to be key to the interpretation of the model, considering the consequences on FQoL that a low family confidence can have. Figure 1 summarizes the path coefficients and illustrates the relationships between the variables.

## 4. Discussion

The scores on caregiver burden showed average levels corresponding to *neutral* burden values with scores similar to those of [36]. With regard to family confidence, we found moderate confidence in both dimensions of the scale, similar to those obtained by [21] in Spain. Finally, the QoL showed acceptable values comparable to previous studies in Spain with the same scale [8,21]. 

In the present study, family confidence was positively related to FQoL, especially the CHF dimension, which was even more related to FQoL than CHC. This result aligns with the findings of [21] with Spanish participants. In addition, other studies pointed out similar constructs to confidence and competence, such as family empowerment, as determining factors and predictors of FQoL [32,42]. 

In our study, caregiver burden negatively predicted family quality of life. Ref. [20] also found these inverse relationships. Although not exactly on QoL, ref. [18] stated that there is a negative relationship between family burden and the “life satisfaction” of parents who have children with disabilities. Specifically, ref. [19] stated that when there is a child with a disability, burden impacts QoL, being a strong negative predictor. 

The child’s support needs did not predict FQoL when analyzed as a predictor, but it was very inversely related to CHC and positively related to caregiver burden. Previous studies have found that severity, the child’s functioning level, and behavioral problems are associated with caregiver burden, lower overall well-being [43,44], increased stress and depression [45], and lower FQoL [14,46]. Ref. [47] did not find an association between severity and burden. Finally, family income was positively and statistically related to both FQoL and family confidence. This result is similar to what [47] pointed out about the relevance of income on the perception of FQoL.

Family confidence appears as a significant predictor that makes other variables, such as income, less relevant. In the presence of family confidence, gender was a significant predictor, indicating that women predicted lower FQoL scores. However, family confidence interacts with sex and shows a positive impact on FQoL, indicating the importance of promoting confidence and competence early childhood intervention services [3].

Finally, this study showed that caregiver burden and child severity, despite being statistically related to lower FQoL scores, when controlling for the effect of family confidence, are no longer statistically significant. It is through low family confidence that these variables can have a negative effect on FQoL. Refs. [14,48] found that severity was associated with lower FQoL scores, and studies in Spain have also confirmed this association [17]. In our study, however, analyzing the severity level in a linear manner has allowed us to examine the indirect influence on FQoL through family confidence, finding this pattern of influence with clear repercussions on services. This result is an indicator of the protective role of helping families develop parental confidence in order to feel competent in helping both their child and the family.

Understanding the impact on FQoL of the parameters related to support services has been studied in Spain in different studies pointing out that services that are more family-centered [8] or more collaborative between families and professionals [12] can be key to improving the quality of family life. While [12] found that both professional–family partnerships and the families’ satisfaction with supports predicted FQoL, in the present study, we focused on the effect of family confidence as one of the empowering outcomes from parent–professional partnerships [49], FQoL. In our study, greater family confidence was key for both improving FQoL and for attenuating the negative impact of caregiver burden and the child’s level of severity. While the effect of caregivers’ self-efficacy beliefs on caregiver burden has been studied in the health and mental health contexts [49,50,51,52], to our knowledge, this relationship has not received as much attention in the field of early childhood intervention.

### 4.1. Limitations

The present study is not without limitations. First of all, it should be noted that the participants in this study were part of ECI centers whose professionals were interested in—or had initiated—an implementation process of recommended, i.e., collaborative family-centered, practices, so the participating families may not be representative of families receiving ECI clinical services with an expert professional approach.

Likewise, the sample size is also a limitation due to the low representativeness. However, both the statistical techniques used—robust and appropriate for these cases—and the pattern of results were similar to those found in the literature relating family confidence to FQoL [21], making this study an interesting contribution. While other studies have analyzed the influence of caregiver burden and family confidence on FQoL separately, our study has focused on burden as the predictor or variable influencing lower FQoL and we analyzed the role of family confidence as a buffer variable against this negative effect. Even with the reduced sample size, the results have pointed in the expected direction and the consequences on support services have a clear direction towards the implementation of collaborative practices and family empowerment. However, this study should be taken as a first attempt in combining all three variables and should be replicated with a larger sample size.

Future studies replicating this pattern of influence could consider analyzing variables that may affect these three variables and their relationship, such as the families’ socioeconomic status, the intervention phase, the time that professionals have been in a process of implementing family-centered practices, or the degree of family-centeredness of the support provided.

### 4.2. Implications

The implications for services that flow from the results of this study focusing on caregiver burden and its effects on FQoL may point in several directions. On the one hand, several authors have pointed out the convenience of support services encouraging families to participate in activities such as mindfulness, which has been shown to be effective in reducing stress in parents with children with disabilities to better cope with the negative experiences that may arise in their day-to-day life with their children [53,54]. In addition, other authors recommend that services provide information to families about support groups, which can contribute to improving parents’ adaptation and their parental competence [48].

Our study provides valuable information by identifying the components of family confidence related to attenuating caregiver burden, as well as identifying the burden and confidence parameters that are most related to FQoL. Specifically, we found that more confidence in helping the family had an influence on both reducing caregiver burden and improving FQoL, even greater than the influence that confidence in helping the child had. These have clear additional implications for services, such as the importance of addressing family issues and adding more family-related goals (goals aimed at the adult caregivers which can be related to the child or not, such as having time for themselves or having information or resources) in the intervention plans in addition to the child-related objectives. This can be crucial in order to reduce the caregiver burden and specifically to develop family competence related to family matters, since it is the dimension of family confidence with a more mediating effect on the relationship between burden and FQoL.

Therefore, the implementation of a family-centered approach and empowering caregivers in their natural environments (i.e., home-based services) and through a primary service provider would be a very suitable combination for adequate family support for improving family confidence and competence, reducing their stress, and seeking a better FQoL.

## Figures and Tables

**Table 1:** Characteristics of participants.

	N	M	SD	Min	Max
Mother–Child Interaction Time	55	4.24	0.98	2	5
Father–Child interaction Time	53	3.26	1.18	1	5
Adult’s Age	58	36.52	9.45	2	56
Child’s age	57	43.42	18.64	3	81
Child’s support needs (1–5)	56	3.43	1.17	1	5
Child’s functioning Level (1–5)	57	4.16	2.99	2	24
Time in ECI	55	16.56	12.49	1	48
	**N**	**%**			
**Family Monthly Income**					
Less than EUR 600	6	11.3			
From EUR 600 to EUR 1200	10	18.9			
From EUR 1200 to EUR 1800	17	32.1			
From EUR 1800 to EUR 2500	7	13.2			
From EUR 2500 to EUR 5000	9	17			
More than EUR 5000	4	7.5			
Did not answer	5	8.6			
Total	58	100			
**Respondent’s relationship with the child**					
Grandmother	2	3.4			
Mother	34	58.6			
Father	10	17.2			
Both Mother and Father completed questionnaire	11	19			
Total	58	100			
**Adult’s Gender**					
Man	14	24.1			
Woman	44	75.9			
Total	58	100			
**Child’s gender**					
Boy	42	72.4			
Girl	16	27.6			
Total	58	100			

**Table 2:** Correlations between continuous variables and burden, confidence, and FQoL.

Variable	1	2	3	4	5	6	7
1. Support needs	—						
2. Severity	0.384 **	—					
3. Income	−0.219	−0.183	—				
4. Burden	0.367 **	0.213	−0.174	—			
5. CHC	−0.461 ***	−0.469 ***	0.412 **	−0.135	—		
6. CHF	−0.334 *	−0.348 *	0.430 **	−0.384 **	0.611 ***	—	
7. FEIQoL	−0.232	−0.362 *	0.359 *	−0.405 **	0.439 **	0.656 ***	—

Note: CHC = confidence in helping the child; CHF= confidence in helping the family. * *p* < 0.05, ** *p* < 0.01, *** *p* < 0.001.

**Table 3:** Model fit for the hierarchical regression models.

					Model’s Global Test			
Model	R	R^2^	R^2^ Adjusted	RMSE	F	df1	df2	*p*
1	0.461	0.212	0.146	0.727	3.231	3	36	0.034
2	0.719	0.516	0.445	0.570	7.262	5	34	< 0.001
3	0.757	0.573	0.495	0.536	7.383	6	33	< 0.001

**Table 4:** Regression coefficients for the prediction of FQoL.

Model	Estimate	SE	t	*p*	β
**Model 1**					
Constant ^a^	3.300	0.536	6.153	<0.001	
Adult Sex (Mothers)	−0.019	0.282	−0.068	0.946	−0.023
Support Needs	−0.143	0.102	−1.410	0.167	−0.214
Income	0.208	0.087	2.396	0.022	0.363
**Model 2**					
Constant ^a^	2.215	0.803	2.760	0.009	
Adult Sex (Mothers)	0.015	0.227	0.067	0.947	0.018
Support Needs	−0.018	0.087	−0.208	0.837	−0.027
Income	0.099	0.076	1.302	0.202	0.173
Burden	−0.395	0.183	−2.162	0.038	−0.286
CHF	0.639	0.197	3.236	0.003	0.461
**Model 3**					
Constant ^a^	4.527	1.344	3.369	0.002	
Adult Sex (Mothers)	−3.020	1.467	−2.059	0.047	−0.047
Support Needs	0.021	0.085	0.246	0.808	0.031
Income	0.136	0.075	1.819	0.078	0.237
Burden	−0.347	0.176	−1.975	0.057	−0.252
CHF	−0.299	0.486	−0.615	0.543	−0.216
CHF × Adult Sex (Mothers)	1.074	0.513	2.093	0.044	0.775

Note: ^a^ Represents the reference level; CHF = confidence in helping the family.

**Table 5:** Parameter estimates of the mediation model.

									95% CI	
Predictor		M		DV	Estimate	SE	z	*p*	Lower	Upper
**Direct effects**										
Burden		→		FEIQoL	−0.358	0.226	−1.584	0.113	−0.802	0.085
Severity		→		FEIQoL	−0.185	0.132	−1.407	0.159	0.073	−0.444
**Indirect effects**										
Burden	→	CHF	→	FEIQoL	−0.286	0.130	−2.203	0.028	−0.541	−0.032
Severity	→	CHF	→	FEIQoL	−0.157	0.072	−2.168	0.030	−0.015	−0.298
**Total effects**										
Burden		→		FEIQoL	−0.645	0.204	−3.158	0.002	−1.045	−0.245
Severity		→		FEIQoL	−0.342	0.138	−2.473	0.013	−0.071	−0.613
**Path coefficients**										
ConFamCAF		→		FEIQoL	0.517	0.119	4.354	<0.001	0.284	0.750
Burden		→		CHF	−0.554	0.206	−2.683	0.007	−0.958	−0.149
Severity		→		CHF	−0.303	0.131	−2.316	0.021	−0.046	−0.559

Note. Robust standard errors, robust confidence intervals, ML estimator; CHF = confidence in helping the family. M = Mediator; DV = Dependent Variable.

## Data Availability

The original contributions presented in the study are included in the article, further inquiries can be directed to the corresponding author/s.
